# A Study of Gene Expression, Structure, and Contractility of iPSC-Derived Cardiac Myocytes from a Family with Heart Disease due to LMNA Mutation

**DOI:** 10.1007/s10439-021-02850-8

**Published:** 2021-09-28

**Authors:** Mehrsa Mehrabi, Tessa A. Morris, Zixuan Cang, Cecilia H. H. Nguyen, Yutong Sha, Mira N. Asad, Nyree Khachikyan, Taylor L. Greene, Danielle M. Becker, Qing Nie, Michael V. Zaragoza, Anna Grosberg

**Affiliations:** 1 Department of Biomedical Engineering, University of California, Irvine, CA 92697 USA; 2 UCI Edwards Lifesciences Foundation Cardiovascular Innovation and Research Center (CIRC), University of California, Irvine, CA 92697 USA; 3 Center for Complex Biological Systems, University of California, Irvine, CA 92697 USA; 4 Department of Mathematics and Developmental & Cell Biology, University of California, Irvine, CA 92697 USA; 5 The NSF-Simons Center for Multiscale Cell Fate Research, University of California, Irvine, CA 92697 USA; 6 Department of Biological Chemistry, School of Medicine, University of California, Irvine, CA 92697 USA; 7 Genetics & Genomics Division, Department of Pediatrics, School of Medicine, University of California, Irvine, CA 92697 USA; 8 Department of Chemical and Biomolecular Engineering, University of California, Irvine, CA 92697 USA; 9 The Henry Samueli School of Engineering, University of California, Irvine, 2418 Engineering Hall, Irvine, CA 92697 USA

**Keywords:** Lamin A/C mutation, iPSC-derived cardiomyocyte, Dysmorphic nuclei, Disease presentation

## Abstract

Genetic mutations to the Lamin A/C gene (*LMNA*) can cause heart disease, but the mechanisms making cardiac tissues uniquely vulnerable to the mutations remain largely unknown. Further, patients with *LMNA* mutations have highly variable presentation of heart disease progression and type. *In vitro* patient-specific experiments could provide a powerful platform for studying this phenomenon, but the use of induced pluripotent stem cell-derived cardiomyocytes (iPSC-CM) introduces heterogeneity in maturity and function thus complicating the interpretation of the results of any single experiment. We hypothesized that integrating single cell RNA sequencing (scRNA-seq) with analysis of the tissue architecture and contractile function would elucidate some of the probable mechanisms. To test this, we investigated five iPSC-CM lines, three controls and two patients with a (c.357-2A>G) mutation. The patient iPSC-CM tissues had significantly weaker stress generation potential than control iPSC-CM tissues demonstrating the viability of our *in vitro* approach. Through scRNA-seq, differentially expressed genes between control and patient lines were identified. Some of these genes, linked to quantitative structural and functional changes, were cardiac specific, explaining the targeted nature of the disease progression seen in patients. The results of this work demonstrate the utility of combining *in vitro* tools in exploring heart disease mechanics.

**Supplementary Information:**

The online version contains supplementary material available at 10.1007/s10439-021-02850-8.

## Introduction

Heart disease, which impacts more than 80 million people just in the USA,^41^ is caused by a variety of factors including genetic mutations.^16,27^ Often the mechanisms by which these mutations cause heart disease are not known,^29,31^ and the mutation is identified purely by studying the genes of large families with a history of heart disease.^6,45,72,73^ Identifying the culprit gene provides relief to the family members who do not have the mutation, but does not usually help the individuals with the mutation. To truly impact such patients, it is essential to elucidate the mechanism linking the mutation to the pathology. Such studies are often possible only with an *in vitro* platform.

There are multiple methods of examining the mutations *in vitro*, but all of them require a source of cells that have the mutation of interest.^1,12,33,37,43,68^ One common way of collecting such cells is to extract skin cells from each patient and to utilize these to make induced pluripotent stem cells (iPSCs), which can then be differentiated into cardiac cells.^1,68^ However, there are many challenges to this approach. First, many inherited mutations do not manifest as heart disease in patients for multiple decades. Thus, for this approach to be viable, it is essential to demonstrate that a pathological phenotype can be recapitulated *in vitro* for this particular mutation. Second, iPSC-derived cardiac cells are notoriously heterogeneous^5,14,71^ creating a challenge when analyzing bulk cell properties, which makes it non-trivial to correlate gene expression to functional phenotypes. If these challenges are successfully addressed, it will be feasible to elucidate possible mechanisms for the heart disease trigger in the patients with the mutation as well as to deepen our understanding of the heart disease cascades. Such information can potentially impact treatment not only in these families but for other patients with unknown heart disease triggers (i.e., no mutation). Indeed, some mutations have correlations with a normal process such as aging.^21^ For example, lamin mutations have correlations with aging in terms of proteins in the nuclear lamina, and variants of these mutations have been extensively studied *in vitro.*^33,39,43,49,63,65^ Many of these studies correlate RNA-sequencing with either structural or functional metrics,^33,49,65^ which provides valuable insight into the possible pathways. However, none of the studies considered anisotropic tissues, which would impact their findings as it has been previously shown that isotropic tissues are weak and have an altered gene expression profile compared to properly organized anisotropic tissues.^19,61^ Furthermore, bulk RNA-sequencing is likely to obscure the heterogeneous nature of the gene expression profiles.^10^

In this manuscript, we present the results of a study of a Lamin A/C (*LMNA*) splice site mutation (c.357-2A>G),^72^ which causes inherited heart disease in patients (Table 1). The patients with this mutation suffer from a variety of heart disease types/symptoms, such as dilated cardiomyopathy (DCM), atrial fibrillation, and heart failure.^42^ Heart disease symptoms can be caused by a variety of concurrent behaviors and mutations, but it is possible to identify specific mutations by considering inherited diseases in multi-generation large families^13,31^ like the one in this study.^72^ In general, *LMNA* mutations can cause a wide variety of heart disease symptoms including the ones suffered by the patients with the splice site mutation studies in this work.^72^ The exact mechanism by which an *LMNA* mutation causes malfunctions in heart structure and function have not been identified. As a result, even though the heart disease causing mutation has been identified in this family, there are no new treatment options for the patients especially because the patients in the family do not have the same symptom presentation and progression among patients can differ by decades (Table 1).^72^ This variability is likely caused by other genes that can protect from or promote the disease progression, which are much harder to identify as they are not conserved through the large family. Therefore, there is a pressing need to create a platform to enable the study of cardiac structure and function with cells genetically identical to any one of the patients on a realistic time-scale. Meeting this need is the main goal of this work. Indeed, here we demonstrate for this mutation that it is possible to construct an *in vitro* platform that has pathology in patient-specific heart tissues when compared to a negative control.

**Table 1:** Individuals’ information involved in the study.

Line	Zaragoza *et al*. symbols^72^	Age of biopsy	First age of presentation of diseases	Symptoms
Patient A1 (PA1)	IV-5	38	36	Bradycardia, premature ventricular contractions, non-sustained ventricular tachycardia, mild dilated cardiomyopathy (DCM), pacemaker
Control A1 (CA1)	IV-2	49	N/A	No Symptoms
Patient A3 (PA3)	III-1	70	49	Bradycardia, atrial fibrillation, pacemaker, dilated cardiomyopathy (DCM), heart failure
Control A3 (CA3)	III-3	68	N/A	No symptoms
Donor 2 (D2)	Normal adult fibroblast	40	N/A	No symptoms

Simultaneously, we show that single cell RNA sequencing (scRNA-seq) analysis can be used to correlate gene expression profiles and functional data to propose possible mechanisms, which potentially trigger pathological heart tissue contractile properties. These results can be used to initiate possible avenues of investigation for (1) intervention for the patients with the mutation and (2) novel mechanisms of heart disease triggers with, for example, aging.

## Methods and Materials

### Substrate Fabrication

Customized coverslips were made for both structural and functional experiments. For structural experiments, a 7.6 cm × 8.3 cm rectangular glass coverslip (Fisher Scientific Company, Hanover Park, IL) was cleaned with 30 min sonication in 200 proof ethanol. The cleaned glass coverslip was then spin-coated with a 10:1 mixture of polydimethylsiloxane (PDMS) and curing agent (Ellsworth Adhesives, Germantown, WI) and cured in 60 °C oven for 12 h. For functional experiments, the glass coverslip was customized according to previously published protocols.^23,24,32^ Briefly, the cleaned glass coverslip was segmented with protective films, was spin-coated with poly (*N*-isopropyl acrylamide) (PIPAAm, Polysciences, Inc., Warrington, PA), and incubated for 5 h at room temperature. The PIPAAm coated glass was spin-coated with a thin layer of PDMS and cured for at least 12 h in the oven. Finally, both versions of coated large glass coverslips (structural and functional) were cut into smaller rectangular coverslips, 14 mm × 12.5 mm, using a diamond scriber (VWR, Radnor, PA). Fibronectin (0.05 mg/mL, Sigma), an extracellular matrix (ECM), was stamped through microcontact printing^32^ on the smaller coverslips in an anisotropic pattern (lines of 20 *µ*m Fibronectin × 5 *µ*m gap). This pattern has been shown to result in engineered constructs that mimic the contractile properties of *ex vivo* heart sections^23,62^ and the electrophysiolgical properties of the heart.^20^ Therefore, through these methods we created patient-specific engineered cardiac tissues.

### Cell Culture

Cell lines used in this project were procured from skin biopsies of four different female individuals (Table 1, PA1, PA3, CA1, and CA3) and a female commercial line [Table 1, D2, Lonza (catalog# CC-2511)]. Skin biopsies were collected as described by Zaragoza *et al*.^72^ The patients have a heterozygous *LMNA* splice-site mutation (c.357-2A>G).^72^ Informed consent of all participants was acquired for this study in accordance with UC Irvine Institutional Review Board (IRB# 2014-1253). All fibroblast lines were reprogrammed with CytoTune-iPS 2.0 Sendai reprogramming kit (ThermoFisher Scientific, Cat# A16517) into iPSCs. The iPSCs were then seeded and passaged on Vitronectin (Gibco, Cat# A1470) or Geltrex (Gibco, Cat# A1413302) coated plates and fed with complete E8 media (Gibco, Cat# A15170) every day for 7 days or until they were 80–90% confluent. Colonies of iPSCs were passaged with ReLeSR (StemCell Technologies, Cat# 05872) to the 15th passage to eliminate the Sendai virus from cultures. Colonies of iPSCs were then singularized with TrypLE (Gibco, Cat# 12604-013) and seeded on Vitronectin or Geltrex coated 12-well plates at the desired density for each cell line. Each cell line was seeded in a range of cell densities ($10\times {10}^{4}$–$50\times {10}^{4}$ cells per well with an interval of 5 × 10^4^ cells per well) in 3 to 4 replicates of each to determine the desired density at which there was maximum cardiomyocytes differentiation efficiency. Thereafter, singularized cells were differentiated with *PSC Cardiomyocyte Differentiation Kit* (Gibco, Cat#A2921201). Usually, on the ninth day of differentiation, beating clusters were observed; however, the cultures were not pure cardiomyocytes. To fix this, cultures were purified with Enriched Cardiomyocytes Media (ECM, Gibco, Pub. # MAN0014509) to substantially reduce the number of non-cardiomyocytes through metabolic selection.^66^ Usually, on the 16th day of differentiation, purified cultures were dissociated with STEMdiff™ Cardiomyocyte Dissociation Kit (StemCell Technologies, Cat# 05025). The cardiomyocytes were then seeded on customized coverslips for functional and structural experiments with supporting cardiomyocyte media from STEMdiff™ kit, and they were fed with M199 media with 10% FBS after 24 h and with 2% FBS after 48 h from seeding day.

### Fixing and Immunofluorescence Staining

After four days of culture on coverslips, the cells were fixed with a solution of 4% paraformaldehyde (Fisher Scientific Company, Hanover Park, IL) and 0.0005% Triton X-100 (Sigma-Aldrich, Saint Louis, MO) for 10 min at room temperature. They were stained for nuclei (4,6-diamidino-2-phenylindole dihydrochloride, DAPI, Life Technologies, Cat# D1306) and various primary antibodies for actin (Alexa Fluor 488 Phalloidin, Life Technologies, Cat# A12379), and *α*-actinin (Mouse Anti-*α*-actinin, Sigma-Aldrich, Cat# A7811). Finally, the primary antibodies were tagged with secondary antibody (Alexa Fluor 630 goat anti-mouse, Life Technologies, Cat# A121050), mounted with Prolong Gold Antifade Mount (Life Technologies, Carlsbad, CA) on to microscope slides, sealed with clear nail polish, and dried overnight.

### Imaging and Image Analysis

The samples were imaged with a Leica SP8 confocal microscope with × 63 (5.54 *µ*m/pixel) oil immersion objective. Ten fields of view were randomly imaged for each sample and analyzed with customized MATLAB software for different features.

#### Nuclei Detection and Evaluation

Nuclei in DAPI stained *z*-stacks were detected by first segmenting each *z*-slice and then grouping nuclei that appeared in multiple *z*-slices. The two-dimensional segmentation was done by first binarizing each *z*-slice using the “CARE” algorithm.^18^ After removing small objects, the watershed transform was performed on the distance transform, which had been modified to filter out tiny local minima.^8,17,44,58^ Individual nuclei that appeared in multiple *z*-slices were grouped by comparing the segmentation results for each neighboring *z*-slice. Finally, the maximum projection of each single nucleus was saved after being approved by the user. The maximum projection of each nucleus was then evaluated by calculating the area, perimeter, eccentricity, maximum negative curvature, mean negative curvature, and relative concavity, as well as classified as normal or dysmorphic, as described previously.^4^

#### Actin Orientation

Each actin stained *z*-slice was enhanced using contrast-limited adaptive histogram equalization.^11^ After enhancing the contrast of each actin stained *z*-slice, the orientation of actin at each pixel was calculated as described previously.^19,23^ Briefly, each image was filtered with a Gaussian kernel and then normalized to have zero mean and unit standard deviation.^34^ The orientation was estimated using a least mean square orientation estimation algorithm.^26,34^ The orientation vectors for each *z*-slice were concatenated for the entire *z*-stack (field of view), and the orientation vectors of each field of view were concatenated for the entire coverslip. Therefore, the total number of actin orientation vectors for a coverslip was calculated by summing the number of orientation vectors contained in each field of view. The orientational order of the actin contained in a coverslip was quantified by the orientational order parameter (OOP), which varies from zero for completely disorganized to one for perfectly aligned vectors and has been described previously.^23,52^

#### Sarcomere Length and Orientation

After the contrast of each *α*-actinin stained *z*-slice was enhanced,^11^ the entire *z*-stack was median filtered.^28^ The sarcomeres in each *z*-slice were detected by first using the “SarcTrack” algorithm, which identifies double wavelets in an image.^22^ The angle of each detected double-wavelet was then compared to the local orientation of actin to remove false sarcomeres, as described in “ZlineDetection”.^7^ The average sarcomere length, sarcomere OOP, and, as for actin, the total number of sarcomeres for each coverslip were calculated after combining all of the *z*-stacks for that coverslip.

### Contractility Measurement

The contractility of the engineered cardiac tissues was assessed using the “heart-on-a-chip” platform, as previously described.^32^ Briefly, the chips were placed in the Normal Tyrode solution, the films were cut out, and the experiments performed at 35–37 °C. The dynamics of the films were recorded using a stereoscope (no. SZX-ILLB2, Olympus America, Center Valley, PA) while the tissues were either allowed to contract spontaneously or were paced using a MyoPacer Field Stimulator (IonOptix, Milton, MA) at 0.5, 1, 1.5, and 2 Hz. The resultant movies were analyzed with custom software to extract diastolic stress, systolic stress, active stress, and beating frequency.^23,32^ The whole dataset is made available through Dryad https://doi.org/10.7280/D10H40.

### Single Cell RNA Sequencing and Analyses

To perform single-cell RNA sequencing analysis, PA1 and CA1 differentiation were done in parallel. The cells were collected using a published protocol (× 10 Genomics, *Sample preparation demonstrated protocol*, Manual part #CG00054) and submitted to UCI genomics high-throughput facility for single-cell RNA sequencing. Seurat package (version 3.1)^47^ was used to analyze the patient and control datasets. The cells not expressing genes *ACTC1* or *TNNT2* and those with over 25% mitochondrial counts were filtered out. We then used the SCTransform utility^48^ in Seurat with default parameters to preprocess the two datasets separately. We next integrated the two datasets using Seurat with the top 3000 variable genes. A UMAP coordination shared by the two datasets were generated using the top 20 principal components. Wilcoxon test was used to determine the globally differentially expressed genes across the datasets.

### Statistics

For structural data, the Student *t*-test was used to compare single pairs of data, and ANOVA with Student–Newman–Keuls *post hoc* test was used for multi-pair comparison. The stress data was determined to be log-normally distributed. Therefore, a *t*-test was used on the log normalized data to compare the patient and control groups.

## Results

**Figure 1:**
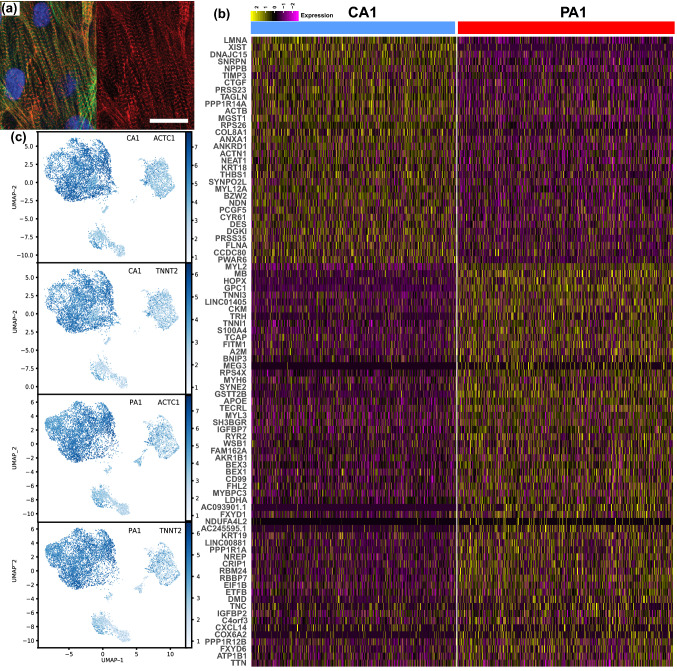
iPSC-derived cardiomyocytes. (**a**) Confocal images of cardiac tissues for one of the individuals. Stained for actin fibrils (phalloidin: green), nuclei (DAPI: blue), and sarcomeric *z*-lines (*α*-actinin: red). Scale bar 20 *μ*m. (**b**) Single cell RNA sequencing of PA1 and CA1 cell lines represented with a heat map for the top 90 genes shows a varied expression profile for the two cells lines. (**c**) Feature maps for the two cells lines show mostly matching clusters for both cell lines, and the two cardiac-specific markers indicate that 99% of the cells are considered cardiomyocytes.

To elucidate the consequences of the *LMNA* splice site mutation (c.357-2A>G),^72^ we first took skin biopsies previously gathered^72^ and created iPSC lines for individuals summarized in Table 1. These iPSC lines were then differentiated into cardiomyocytes and engineered to be anisotropic cardiac tissues (Fig. 1a). To ensure that the structural and functional data could be correlated to gene expression variations, one pair (PA1 and CA1) were analyzed with scRNA-seq (Fig. 1b, Supplemental Data File 1). The cardiac tissues were confirmed to have sarcomeres by staining *z*-lines with an *α*-actinin stain, imaging, and confirming the presence of striations (Fig. 1a). Additionally, scRNA-seq showed that on average 99% of cells were positive for Troponin 2 (*TNNT2*) and Actin Alpha Cardiac Muscle 1 (*ACTC1*) cardiac markers (Fig. 1c). As a result, the expression levels for the genes of interest were essentially identical for the full data set and the data set of cells expressing standard *TNNT2* and *ACTC1*, which are considered classical cardiac markers (Supplemental Data Files 1 and 2). Interestingly, the clusters for the patient cell line (PA1) and the control cell line (CA1) look similar (Fig. 1c), and there are no unique clusters in PA1 that do not exist in CA1 or vice versa. Driven by the mutation, *LMNA* is differentially expressed between the patient and control cells (Fig. 1b). The other differentially expressed genes can be of great interest in identifying mechanistic differences that could potentially be triggered by the mutation, and the most interesting ones are discussed along with the structural and functional in the following sections of these results.

**Figure 2:**
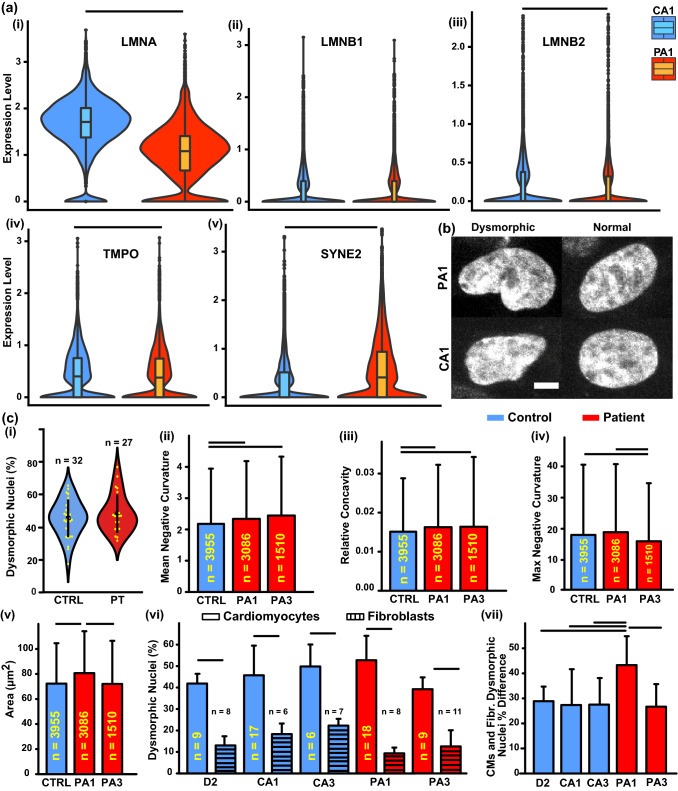
Nuclei analysis. (**a**) Nuclei related gene expressions from single cell RNA-sequencing of two cell lines—PA1 (cell number = 13,028) and CA1 (cell number = 12,591). (**b**) Example images of normal and defective nuclei, stained for DAPI, for a patient and control cell line. Scale bar 5 *μ*m. (**c**) Quantitative analysis of nuclear morphology for all five cell lines (error bars are standard deviations; black horizontal lines indicate significance of *p* < 0.05). Unless otherwise indicated sample sizes (*n*) is the number of nuclei analyzed for each condition: (i) percent dysmorphic nuclei grouped by mutation status—controls (CTRL) and patients (PT) (sample sizes, *n* based on the number of coverslips). (ii) Mean negative curvature, (iii) relative concavity, (iv) maximum negative curvature, and (v) area for all controls and for each individual patient line. (vi) Comparison of dysmorphic nuclei percentage for both cardiomyocytes and fibroblasts in all individual cell lines (sample sizes, *n* based on the number of coverslips). (vii) The increase in dysmorphic nuclei percentage from fibroblasts to cardiomyocytes for each individual [errors were propagated from part (vi) and smaller sample size was used for each comparison].

Variants of *LMNA* mutations are known to adversely impact the nuclear shape.^3,51^ To investigate this possibility for the (c.357-2A>G) mutation, the scRNA-seq data for nuclei lamina-related genes was analyzed. The findings align with the identified mutation^72^ with a significantly lower expression of *LMNA* for PA1 compared to CA1 [Fig. 2a(i)]. Other lamin proteins, *LMNB1* [Fig. 2a(ii)] and *LMNB2* [Fig. 2a(iii)], did not have as much of a difference even though *LMNB2* was statistically significant. Out of the proteins that interact with *LMNA* directly and help to maintain nuclei structural integrity, there was a small but statistically significant difference in *TMPO*, coding for *LAP2* protein, and a larger difference in *SYNE2*, coding for Nesperin-2. Interestingly, *TMPO* is expressed at slightly lower levels in the patient line, which is, on its own, known to be associated with the DCM in patients.^25^ By contrast, *SYNE2* has higher expression levels in the patient line, indicating that there are compensation mechanisms. Thus, to understand how this gene expression landscape emerges into cell and tissue phenotype, the nuclei of the cardiomyocytes were stained with DAPI and imaged via a confocal microscope (Fig. 2b). Each nucleus was extracted from the image stack using customized code and analyzed with previously developed metrics^4^ (Fig. 2c). These metrics included the mean negative curvature [Fig. 2c(ii); Fig. SF4.1] and the relative concavity [Fig. 2c(iii); Fig. SF4.2], both of which represent the average amount by which the surfaces of the nuclei bend inward with the former sensitive to sharp defects and the latter focused on the overall concave area. The other metrics included the maximum negative curvature of the perimeters of the nuclei [Fig. 2c(iv); Fig. SF4.3], which partially characterizes the degree of defect, and the area of the nuclei [Fig. 2c(v); Fig. SF4.4], which can also change as nuclei deform. These parameters were used to classify the fraction of dysmorphic nuclei as previously described^4^ [Fig. 2c(i) and (vi)]. For this data 32 and 27 cover-slips with cells from control and patient lines, respectively, were analyzed for the proportion of the defective nuclei. The data can be analyzed for each cell line that comes from a specific individual (Table 1, Supplemental Data File 4) or it can be pooled to consider all tissues made from cells with a mutation (PT) or without a mutation (CTRL). The latter approach is important when considering the general effect of the mutation, while the former is appropriate to ensure that any one individual is not driving the overall difference. This results in a large number of permutations of how the data can be analyzed, therefore the raw data is made available, the Supplemental Data File 4 have a number of figure permutations that the reader might find informative, and here we present the most interesting findings. While there was no statistical significance if the means of the percentage of the defective nuclei for the two categories (PT vs. CTRL) were compared [Fig. 2c(i)], the proportion of the defective nuclei in the control (5116:11,696) was statistically less than the proportion in the patient category (3601:7695) based on a *z*-test with *p* < 0.001. Further, it appears that PA1 dysmorphic nuclei have more severe defects (Fig. 2b) quantitatively indicated by greater nuclear mean negative curvature [Fig. 2c(ii); Fig. SF4.1], relative concavity [Fig. 2c(iii); Fig. SF4.2], maximum negative curvature [Fig. 2c(iv); Fig. SF4.3], and nuclear area [Fig. 2c(v); Fig. SF4.4]. Analysis of PA3 nuclei showed some indicators of more severe defects like the mean negative curvature [Fig. 2c(i)] and relative concavity [Fig. 2c(ii); Fig. SF4.1], but the other indicators did not follow the same trend as PA1 [Fig. 2c(iv, v); Fig. SF4.3, 4]. These findings align with PA3 developing heart disease much later in life than PA1. The percent of dysmorphic nuclei was also compared for each individual between fibroblasts and cardiomyocytes [Fig. 2c(vi)].^4^ As expected,^25^ for each individual the amount of dysmorphic nuclei was significantly higher in cardiac tissue than in fibroblasts. The increase itself was significantly greater for PA1 [Fig. 2c(vii)] who developed heart disease at an early age than for the other cell line (Table 1).

**Figure 3:**
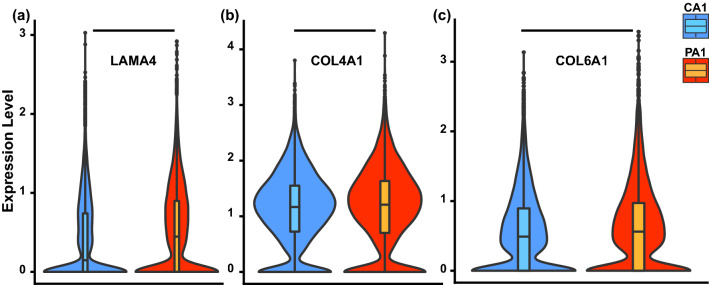
Extracellular matrix. Extracellular matrix gene expression levels for CA1 (cell number = 12,591) and PA1 (cell number = 13,028) cell lines for (**a**) Laminin subunit Alpha 4 (*LAMA4*), (**b**) Collagen type IV Alpha 4 (*COL4A1*), and (**c**) Collagen type VI Alpha 1 (*COL6A1*).

There has been evidence that in heart failure, the ECM undergoes remodeling that contributes to further pathological changes.^9,64^ The ECM is a conduit for the mechanotransduction between the cell nucleus and the outside environment. Therefore, the ECM composition can affect both cell function and further ECM generation.^3^ For examining the effect of LMNA mutation on probable ECM composition, the scRNA-seq^30^ data were compared between patient and control cells for the expression of ECM proteins known to be indicative of heart disease.^9^ Twelve ECM genes from the family *TIMP*, *MMP*, and *COL#A1* were found to be overexpressed in patient cells indicating possible essential consequences of the mutation (example shown in Fig. 3, all provided in Supplemental Data File 3). However, three of the genes from these families were found to be overexpressed in the control cells compared to the patients (Supplemental Data File 3). The overexpression of the ECM proteins can make the environment stiff and affect the function of the cardiomyocytes including decreasing systolic stress [Fig. 5e(ii)], which is one of the DCM symptoms.^30^

**Figure 4:**
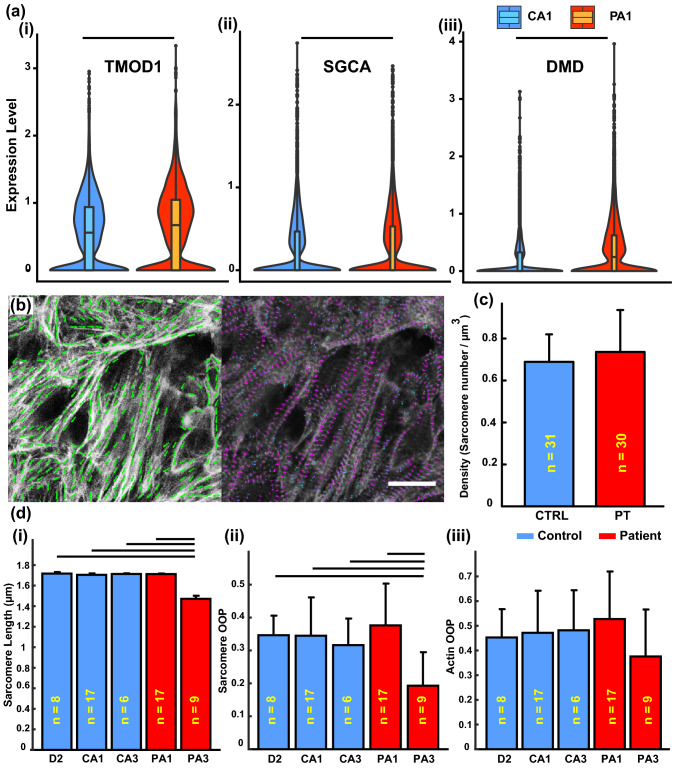
Cardiomyocyte cytoskeleton. (**a**) Expression levels for cytoskeleton related genes that were differentially expressed in CA1 (cell number = 12,591) and PA1 (cell number = 13,028) lines. (**b**) Example analysis of actin orientation (left image, green arrows) and sarcomere identification [right image sarcomeres identified with purple (longer) and blue (shorter)]. (**c**) The density of sarcomeres averaged for three control lines (CTRL) and two patient lines (PT). (**d**) Quantification of myofibril architecture (error bars are standard deviations; black horizontal lines indicate significance of *p* < 0.05): (i) average sarcomere length for each individual cell line; orientational order parameter (OOP) for the (ii) sarcomeres and (iii) actin. Sample sizes (*n*) are based on the number of coverslips analyzed for each condition.

The next series of genes code for a variety of cytoskeleton proteins (Fig. 4a). There was a statistically significant difference between PA1 and CA1 for *TMOD1*, *SGCA*, and *DMD*; all of which had higher expression in the patient cells (Fig. 4a). *TMOD1* codes for a member of the tropomodulin family of Proteins, which are responsible for regulating tropomyosin and inhibiting depolymerization and elongation of the pointed end of the actin.^36^ Consequently, an increase in the expression of *TMOD1* can result in shortened sarcomeres. Alternatively, overexpression of *TMOD1* can lead to degeneration of the myofibrils, which is associated with some clinical presentations of cardiomyopathies.^40^ It is important to note that many cells in the patient populations have the same expression level of *TMOD1* as the control cells, but there are more cells with a higher expression in PA1 leading to the higher average expression [Fig. 4a(i)]. The other two genes, *SGCA* and *DMD*, that are overexpressed in PA1 code for proteins that bind together and stabilize the muscle fibers and prevent injury during the contraction cycles.^38,53,60^ These results indicate that the emergent phenotypes for the patients might be highly variable depending on whether the distractive or protective factors dominate.

To examine the emergent cardiac cytoskeleton architectures, the tissues were stained for actin and *z*-lines (*α*-actinin) (Fig. 1a). The images were analyzed by customized code (Fig. 4b). As we are ultimately interested in comparing the force production capability of the tissues made from cells with or without a mutation, it was essential to ensure that the number of contractile units in these groups were the same. Indeed, the analysis indicated that the number of sarcomeres per unit volume of cardiac tissue is the same for patient and control lines (Fig. 4c; Fig. SF4.5). However, one of the patient lines (PA3) has significantly shorter sarcomeres compared to all of the other individuals, and its sarcomeres were not as well organized [Fig. 4d(i)–(ii); Fig. SF4.6].

**Figure 5:**
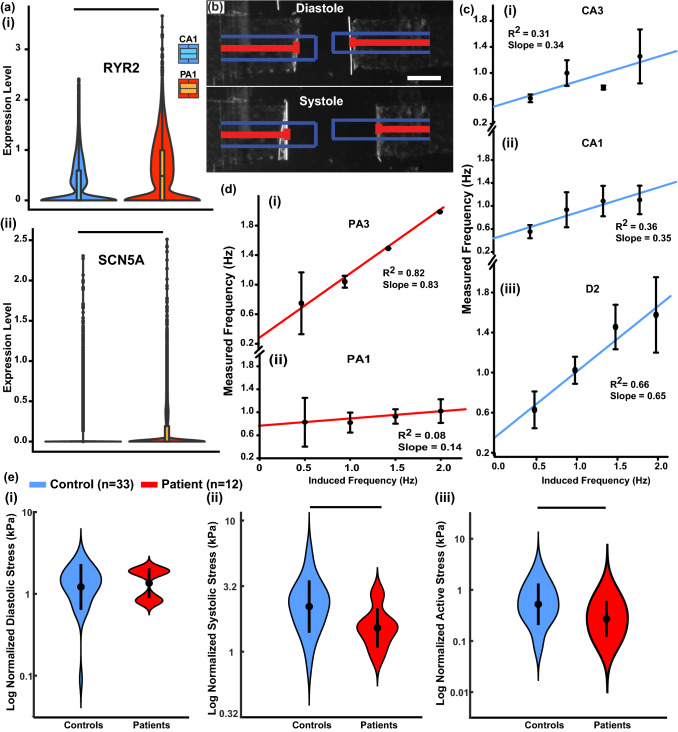
Contractility of cardiac tissues. (**a**) Expression levels for genes, which contribute to contractile properties, differentially expressed for PA1 (cell number = 13,028) and CA1 (cell number = 12,591). (**b**) An image of a “heart-on-a-chip” device for systole and diastole. Blue outlines the length of the film when it lies flat, and red bars track the horizontal projection of the films that is used to calculate stress as a function of time. Scale bar 1 mm. Measured beating frequency vs. pacing frequency with a linear regression for (**c**) control tissues (sample size Table S1) and (**d**) patient tissues (sample size Table S1). (**e**) Log normalized Diastolic (i), Systolic (ii), and Active (iii) stresses averaged for the three control and two patient lines shows a statistically significant difference for systolic and active stresses.

Based purely on structural findings, PA1 had more nuclear defects, while PA3 had sarcomere architecture problems. Either of these could lead to changes in functional properties. Therefore, the genes associated with electrophysiology and known contractility properties were analyzed next. Two genes were overexpressed by PA1 cells, *RYR2* and *SCN5A* (Fig. 5a). *RYR2* is a calcium channel protein that dysregulates calcium transition when overexpressed, which decreases the systolic contraction of the ventricle.^70^ The *SCN5A* codes for a sodium channel protein, and its overexpression leads to the shorter P-wave duration and P–R intervals,^2,59^ which implies a shorter period for diastolic filling. These findings indicate a possibility of functional pathologies within the myocardium. To test this, the “Heart-on-a-chip” platform was used to measure contractile properties in all five individuals (Fig. 5b). Due to the stem cell origin of the cells in the tissues, there was inconsistency in tissues’ response to induced pacing (Figs. 5c and 5d), which made it challenging to relate the finding to the gene expression data. However, when considering generated stress, it was possible to compare tissues with and without the mutation. Indeed, tissues that were beating at approximately 1 Hz, were statistically significantly weaker in the systolic and active stress generation for patient versus control groups (Fig. 5e). While the sample sizes are not large enough if each individual cell line is considered separately, it consistently trends with the patients having lower stresses than the controls even if the stress data for each individual cell line is normalized by the average sarcomere density for that cell line (Figs. SF4.12 and SF4.13). At this frequency, there was no statistically significant difference between the rise and fall time of the contraction, which is not surprising when comparing contractions with the same frequency. Other contraction modalities could potentially be analyzed for differences in dynamics in the future, so we provide any interested reader with access to the raw data. Interestingly, the stress measurements were log-normally distributed for both control and patient lines indicating that the mechanisms influencing contraction strength multiply in effect. Additionally, it is curious that there was no statistically significant difference between PA1 and PA3 stress generation (Figs. SF4.6, SF4.7, SF4.8, SF4.9, SF4.10, SF4.11, SF4.12 and SF4.13 and raw data link provided in Methods section) even though the type of structural defects were different for the two patients.

## Discussion

As the main deliverable in this work, we have demonstrated that even though (c.357-2A>G) mutation does not manifest in patients as heart disease for multiple decades (Table 1),^72^ the *in vitro* platform makes it possible to study pathological function (Fig. 5e; Figs. SF4.6, SF4.7, SF4.8, SF4.9, SF4.10, SF4.11, SF4.12 and SF4.13) within a few weeks of differentiation. The functional difference is statistically significant even though the stem cell-derived cardiomyocytes are highly heterogeneous for each cell line. These heterogeneities lead to many samples with artificially low stress generation, which requires relatively high sample sizes to differentiate between experimental groups. However, because the two groups had on average a similar number of sarcomeres in a given volume, the ability of the engineered tissues with no mutations to generate higher stresses is indicative of a healthier contractile mechanism. For each group, both the stronger and weaker tissues were included in the comparison to ensure no bias in result interpretation. The log-normal distribution of stress generated by iPSC-derived cardiomyocytes probably arises from the total stress being the multiplication of such factors as sarcomere density, which is highly variable locally (Figs. 4b and 4c), sarcomere length, and the amount of force produced by each sarcomere. This type of data could significantly aid in future models of iPSC-derived cardiomyocyte contractility.^32^ As an approach to combat the uncertainty arising from large heterogeneities, we have also demonstrated the power of combining scRNA-seq analysis with functional data and quantitative metrics extracted from three-dimensional confocal images of highly heterogenous stem cell-derived cardiac tissues.

In the last several years, there have been multiple investigations of the pathways by which *LMNA* mutation could potentially cause a variety of heart disease symptoms.^33,39,43,46,49,50,55,56,63,65,67^ The varied approaches were able to elucidate the effect of the mutations on apoptosis,^63^ electrophysiology,^33,65^ sarcomere alignment,^33,39^ and qualitative tissue architecture.^33,39,49,63^ However, as we demonstrated (Fig. 5; Figs. SF4.6, SF4.7, SF4.8, SF4.9, SF4.10, SF4.11, SF4.12 and SF4.13), multiple factors contribute to the emergent tissue force generation, which is the function that best correlates with the clinical ejection fraction of the left ventricle. These works identified multiple pathways by which the mutation could affect the heart, such as PDGF pathway^33^ and ERK1/2 pathway.^49^ However, these investigations did not correlate the variable gene expression landscapes to the emergent contractile force generation of the tissue, and thus it is likely that more pathways are involved than those identified in the prior works. Some of these papers performed bulk RNA-sequencing, which elucidated the gene expression level for whole tissue in culture.^33,49,65^ Nevertheless, the lack of single cell gene expression resolution means that it is unclear if all the cells with the mutation have a different gene expression level or if there are variable populations of cells. As a result, the identification of any single pathway of disease progression is likely to miss concurrent pathologies.

Single-cell RNA sequencing is a powerful method for investigating cell populations.^71^ A possible hypothesis for the progression of disease in this family was that the patients have a small cell population in their hearts that would lead to the clinical symptoms when the pathological cell number exceeded some critical level. Alternatively, it was possible that the cardiac cells were naturally more susceptible to damage because of the mutation. One of the important results of this work is that the scRNA-seq data demonstrates that there are no unique clusters in the patient line (Fig. 1c) even though there are structural (Figs. 2b–2c, 4c–4d) and functional (Fig. 5e; Figs. SF4.6, SF4.7, SF4.8, SF4.9, SF4.10, SF4.11, SF4.12 and SF4.13) differences from the control line. This provides evidence in favor of the second hypothesis that could potentially be confirmed by further studying cardiac samples from the patients if those were ever available. This type of a conclusion would have been impossible with bulk RNA sequencing. In the future, this approach can also be utilized to look at other families with similar mutations^15,72^ as well as sex as a biological variable. This would require significantly more individuals to be analyzed with the assays detailed here, which was beyond the scope of this study. An alternative approach could be to create isogenic lines by introducing a mutation to a control line.^54,69^ While this powerful approach was beyond the scope of the current study, it could isolate the pathway triggered specifically by the LMNA mutation. However, in such an approach the variability between family members would be lost unless the patients with variable presentations were each used to make an isogenic control with corrected LMNA gene.

One of the big motivators behind this study was to pinpoint the possible mechanisms for the initiation and progression of the pathology in the patients. Our integrated approach demonstrated that there are competing factors leading to the wide distribution of symptoms in the patients. For example, the higher expression of *TMOD1* could lead to either shortening of sarcomeres or the degeneration of myofibrils during contraction.^36,40^ The identification of *TMOD1*, a cardiac specific isoform, as one of the culprits of the loss of function would also explain why the patients in this family have no skeletal muscle pathology. However, complicating the story, there are possible compensating mechanisms that are also triggered, which are indicated by the higher expression of *SGCA* and *DMD* (Fig. 4a). These competing factors could explain why patients with the same mutation could have such different disease presentations.^4,45,57,72^

Similar compensation mechanisms are seen in the genes responsible for the nuclear shape and structural integrity. Indeed, even though the *LMNA* expression level is significantly lower in the patient than control cells, *SYNE2*, which has a stabilizing effect on the nuclear structural integrity, has a higher expression in the patient cells. This type of compensation mechanism could explain the thirteen-year difference in the initial presentation of the disease (Table 1).

Minor nuclear defects are a normal feature of nucleated cells. It has been shown that the amount of dysmorphic nuclei increases with age,^4^ and that cardiac cells have more dysmorphic nuclei than skin fibroblasts,^25^ which is also confirmed by our data [Fig. 2c(vi)]. It is curious that if the amount of dysmorphic nuclei is taken as a symptom of aging, some individuals seem to age slower than others.^4^ Indeed, we have previously shown^4^ that PA3 fibroblasts, unlike PA1 fibroblasts, exhibit significantly fewer dysmorphic nuclei than would be expected for their respective age groups. It is possible that in PA3, the protective mechanisms help maintain nuclear structural integrity better than PA1, which is born out in the quantification of nuclear defects (Fig. 2c; Figs. SF4.1, SF4.2, SF4.3 and SF4.4). Furthermore, if the number of dysmorphic nuclei in the cardiac tissue is compared between age-matched pairs, PA1 cardiomyocytes have significantly more defective nuclei than D2 cardiomyocytes. In contrast, PA3 cardiomyocytes have fewer defective nuclei than CA3. In sum, these results point to individual-to-individual variability, which makes experiments with patient-specific cells a critical component of these studies.

In cardiomyopathies, the ECM is often remodeled^9,64^ changing the mechanical environment in which the cardiomyocytes are contracting. In light of this, the correlation between the symptoms of the patients with DCM and the higher expression of ECM proteins associated with DCM (Fig. 3) hints at another pathway that could be detrimentally affecting contraction in the patients who clinically are known to have DCM. Indeed, one of the avenues of further study uncovered by this work, is the differential ECM expression that could potentially be triggered by the mutation (Table 2). However, this will require more cell line characterization because even though the vast majority of the cells were positive for the classical cardiac markers, there were cells in each tissue that did not develop mature striated sarcomeres, and it is possible that the variability in the differentiation process leads to variations in the ECM composition produced by the cells. Such variation would be averaged out for all the structural and functional experiments presented here, but further study would be needed to fully identify which cardiac cell linages have the higher expression of the relevant ECM protein (Table 2). Still, from the findings of all the abnormal expression levels in genes that can potentially lead to cardiac abnormalities, i.e., structural, ECM, and electrophysiological, it is clear that these patients could be entering the heart disease cascade through slightly different mechanisms, all of which originate at the *LMNA* mutation. However, the one consistent emergent property of tissues engineered from patient stem-cell derived cardiomyocytes was that they generate significantly less stress than non-mutated tissues (Fig. 5e; Figs. SF4.6, SF4.7, SF4.8, SF4.9, SF4.10, SF4.11, SF4.12 and SF4.13).

**Table 2:** Single cell RNA-seq for additional ECM proteins.

ECM genes	Over expressed in:	Significant/not significant
TIMP3	CA1	Significant
TIMP2	CA1	Significant
TIMP4	PA1	Significant
TIMP1	PA1	Not significant
MMP23B	PA1	Significant
MMP9	PA1	Significant
MMP1	CA1	Significant
MMP24OS	PA1	Significant
MMP15	PA1	Significant
MMP25-AS1	PA1	Significant
MMP2	PA1	Significant
MMP14	PA1	Significant
MMP24	PA1	Significant
MMP16	CA1	Significant
MMP28	PA1	Significant
MMP3	CA1	Not significant
MMP21	PA1	Not significant
MMP10	PA1	Not significant
MMP11	CA1	Not significant
MMP17	CA1	Not significant
MMP19	CA1	Not significant
COL18A1	PA1	Significant
COL3A1	PA1	Significant
COL1A1	PA1	Significant
COL15A1	PA1	Not significant

### Conclusion

There are varieties of LMNA mutations, which have heart disease as the only devastating pathology.^35,72^ It has been a mystery how a mutation to the nuclear lamina, which should manifest in all nucleated cells, only affects the heart. Further, many patients with these mutations have widely varying symptoms of presentation. Here we demonstrated an integrative method that elucidated the multiple possible mechanisms through which the disease could be initiated. None of the methodologies on their own would have been able to show the intricate connection among gene expression, cell and tissue structure, and cardiac tissue function. Our approach yielded a platform that can be used for further studies of these mutations and illustrated the importance of evaluating patient-specific tissues. In the future, this approach can be made even more potent by considering more individuals with and without the mutation.

## Supplementary Information

Below is the link to the electronic supplementary material.

Supplementary file1 (DOCX 26 kb)

Supplementary file2 (DOCX 17 kb)
