# Supplementary material for: A Study of Gene Expression, Structure, and Contractility of iPSC-Derived Cardiac Myocytes from a Family with Heart Disease due to LMNA Mutation
Source: Ann Biomed Eng. 2021 Sep 28;49(12):3524–39. doi: 10.1007/s10439-021-02850-8 (PMC8671287; doi:10.1007/s10439-021-02850-8)
Supplement: Supplementary file 1 — Supplementary file1 (DOCX 26 kb) [file 10439_2021_2850_MOESM1_ESM.docx]

Table S1: Sample sizes for Heart chip data in Fig 5C and Fig 5D

| **Sample Name** | **Induced Frequency (Hz)** | **Sample Size** |
| --- | --- | --- |
| **D2** | **0.5** | **11** |
| **D2** | **1** | **12** |
| **D2** | **1.5** | **11** |
| **D2** | **2** | **12** |
| **CA1** | **0.5** | **11** |
| **CA1** | **1** | **12** |
| **CA1** | **1.5** | **13** |
| **CA1** | **2** | **13** |
| **CA3** | **0.5** | **7** |
| **CA3** | **1** | **8** |
| **CA3** | **1.5** | **8** |
| **CA3** | **2** | **9** |
| **PA1** | **0.5** | **7** |
| **PA1** | **1** | **8** |
| **PA1** | **1.5** | **8** |
| **PA1** | **2** | **8** |
| **PA3** | **0.5** | **4** |
| **PA3** | **1** | **4** |
| **PA3** | **1.5** | **4** |
| **PA3** | **2** | **4** |
