# Supplementary material for: A Study of Gene Expression, Structure, and Contractility of iPSC-Derived Cardiac Myocytes from a Family with Heart Disease due to LMNA Mutation
Source: Ann Biomed Eng. 2021 Sep 28;49(12):3524–39. doi: 10.1007/s10439-021-02850-8 (PMC8671287; doi:10.1007/s10439-021-02850-8)
Supplement: Supplementary file 2 — Supplementary file2 (DOCX 17 kb) [file 10439_2021_2850_MOESM2_ESM.docx]

**Supplemental Files** for “A Study of Gene Expression, Structure, and Contractility of iPSC-Derived Cardiac Myocytes from a Family with Heart Disease due to LMNA Mutation” can be found at:

**Links:**

Zenodo: <https://doi.org/10.5281/zenodo.4784756>

Dryad: <https://doi.org/10.7280/D10H40>

**Supplemental File 1**

This is an excel file that contains the raw data from single cell RNA-seq experiments. This is part of raw data that will be downloaded as part of the doi.

**Supplemental File 2**

This file contains figures for every gene presented in Fig. 2-5 showing no substantial difference between filtered and non-filtered single cell RNA-seq analysis. This file can be downloaded from the link in Zenodo (same link).

**Supplemental File 3**

RNA-seq single cell results for additional ECM genes of interest – a complement to Figure 3 and Table 2. This file can be downloaded from the link in Zenodo (same link).

**Supplemental Data (File 4)**

Supplemental file containing complementary Figures showing the data broken down by individual cell line. This file can be downloaded from the link in Zenodo (same link).

The doi link also contains the raw data associated with the MTF and structure.
